# Diabetes, bone and glucose-lowering agents: basic biology

**DOI:** 10.1007/s00125-017-4269-4

**Published:** 2017-04-22

**Authors:** Beata Lecka-Czernik

**Affiliations:** 1 Departments of Orthopaedic Surgery, MS 1008, Health Sciences Campus, The University of Toledo, 3000 Arlington Avenue, Toledo, OH 43614 USA; 2 Physiology and Pharmacology, Health Sciences Campus, The University of Toledo, Toledo, OH USA; 3 Center for Diabetes and Endocrine Research, Health Sciences Campus, The University of Toledo, Toledo, OH USA

**Keywords:** AGEs, Bone quality, Bone remodelling, Bone vasculature, Glucose, Glucose-lowering therapies, Muscle, Osteoblast, Osteoclast, Review, Stem cells

## Abstract

Skeletal fragility often accompanies diabetes and does not appear to correlate with low bone mass or trauma severity in individuals with diabetes. Instead (and in contrast to those with osteoporotic bone disease), bone remodelling and bone turnover are compromised in both type 1 and type 2 diabetes, contributing to defective bone material quality. This review is one of a pair discussing the relationship between diabetes, bone and glucose-lowering agents; an accompanying review is provided in this issue of *Diabetologia* by Ann Schwartz (DOI: 10.1007/s00125-017-4283-6). This review presents basic science evidence that, alongside other organs, bone is affected in diabetes via impairments in glucose metabolism, toxic effects of glucose oxidative derivatives (advance glycation end-products [AGEs]), and via impairments in bone microvascular function and muscle endocrine function. The cellular and molecular basis for the effects of diabetes on bone are discussed, as is the impact of diabetes on the stem cell niche and fracture healing. Furthermore, the safety of clinically approved glucose-lowering therapies and the possibility of developing a single therapy that would be beneficial for both insulin sensitisation and diabetes bone syndrome are outlined.

**Electronic supplementary material:**

The online version of this article (doi:10.1007/s00125-017-4269-4) contains a slideset of the figures for download, which is available to authorised users

## Introduction

Skeletal fragility often accompanies type 1 and type 2 diabetes and is considered a pathological complication of this disease. Although low bone mass in type 1 diabetes may significantly contribute to an increase in fracture risk, an increase in fracture incidence is also observed in individuals with type 2 diabetes despite normal or even high bone mineral density (BMD) and greater BMI (factors that are considered protective against fractures in individuals without diabetes, as discussed further in an accompanying review by Ann Schwartz, in this issue of *Diabetologia* [1]). Similarly, fractures that often result from low or medium trauma cannot be explained by increased falls as a result of diabetes-related comorbidities [2]. Instead, despite high/normal BMD, in individuals with diabetes bone shows a number of structural characteristics predisposing it to fractures, including greater cortical porosity, smaller cortical area and decreased bone material strength [3, 4]. These features suggest that bone biomechanical quality is affected in individuals with diabetes.

Bone material quality is maintained by the process of bone remodelling, which is common for all mammals. Bone remodelling relies on constant resorption and rebuilding of bone in order to replace old tissue with new, more functional tissue. A balance between osteoclast-dependent bone resorption and bone formation, the latter of which relies on osteoblast activity, is essential for the maintenance of bone mass. Consequently, most metabolic bone diseases, including senile and postmenopausal osteoporosis, result from unbalanced bone remodelling. On the contrary, bone in diabetes is characterised by attenuated (rather than unbalanced) bone remodelling. Interestingly, decreased bone formation, as measured in iliac bone biopsies, was found to correlate with duration of diabetes [5] and circulating levels of biochemical markers of bone formation and resorption are shown to be decreased in diabetes [6]. It is speculated that low turnover of bone in diabetes may lead to defective microfracture repairs and, hence, to their accumulation, contributing to decreased bone quality. In contrast to postmenopausal and senile osteoporosis, a deterioration of bone strength in diabetes is associated with increased cortical porosity that is not accompanied by a loss of trabecular bone mass [7, 8]. Thus, it can be concluded that diabetes-specific bone characteristics may constitute a novel syndrome that can be classified as a diabetes-associated bone disease. This review addresses the skeletal consequences of the major features that characterise diabetes, including impairments in glucose/insulin metabolism, accumulation of advanced glycation end-products (AGEs), insufficiency of the bone microvasculature and alterations in muscle endocrine function. Alongside the pleiotropic effects of these factors on bone degeneration, the impact of glycaemic control in response to existing glucose-lowering therapies is discussed; an overview of the clinical safety of glucose-lowering therapies is provided in the accompanying review [1], whereas this review focusses on the laboratory evidence of the effects of these therapies on bone.

## Insulin signalling, glucose metabolism and bone turnover

Energy metabolism and bone turnover are controlled by intricate mechanisms that share many cues and outcomes. Because of the size of the skeleton and the extent of energy consumed during the process of bone remodelling, the skeleton is an organ of the body that heavily depends on glucose metabolism. Therefore, it is not surprising that insulin signalling plays an important role in the regulation of bone remodelling. More specifically, osteoblasts require glucose for differentiation and function [9], while glucose and insulin positively regulate the expression of runt-related transcription factor 2 (RUNX2) and bone-specific osteocalcin, the latter being a hormone that is implicated in the regulation of insulin sensitivity in peripheral tissues [10]. In addition, insulin increases support for osteoclastogenesis by decreasing the expression of osteoprotegerin, a decoy receptor for the pro-osteoclastic cytokine receptor activator of nuclear factor κB ligand (RANKL) [11]. Interestingly, the same system has recently been implicated in the regulation of insulin production in pancreatic beta cells, providing additional evidence that bone and energy metabolism are regulated by closely related mechanisms [12]. Taking into consideration that insulin signalling and glucose metabolism correlate positively with bone turnover and bone formation, a logical question arises as to whether bone develops insulin resistance and, if so, how it is manifested.

Some answers come from extensive studies in rodent models where mimicking insulin resistance in bone by depleting insulin receptor in cells of osteoblast lineage results in decreased bone formation and bone resorption and, as a consequence, decreased bone turnover [11, 13]. In turn, manipulation of insulin signalling at the osteoblast level affects systemic energy metabolism. The postulated mechanism implicates bone turnover as a factor that controls energy balance through release of the bioactive form of osteocalcin from the mineralised matrix, which in turn stimulates insulin secretion [10]. Comparably, glucose intolerance is associated with the attenuation of bone remodelling and turnover in mouse models of diet-induced obesity (DIO) [14–16]. Interestingly, analogous with adult onset of type 2 diabetes, the development of DIO in adult mice that have achieved peak bone mass results in high bone mass and attenuated bone turnover [16]. The increased bone mass that accompanies DIO may reflect a protective mechanism, emulating the cessation of bone turnover that provides protection from bone loss that naturally occurs during ageing; however this mechanism does not seem to protect from the loss of bone material quality. Most importantly, osteoblasts in mice with DIO exhibit characteristics of insulin resistance as they do not respond to the stimulatory effect of insulin on the phosphorylation of IRS1/2. Saturated fatty acids and associated lipotoxicity appear to be culprits for the dysfunctional insulin signalling in bone from animals with DIO [14].

### Molecular mechanisms linking bone homeostasis with glucose metabolism

At the molecular level, skeletal homeostasis is linked to insulin sensitivity through the nuclear receptor peroxisome proliferator activated receptor (PPAR)γ. Most recently, it has been shown that the same post-translational modifications of the PPARγ protein that regulate insulin sensitivity and energy metabolism also regulate bone turnover, providing an insight into the intricate relationship between these processes [17]. PPARγ controls differentiation of cellular components of bone remodelling; it suppresses osteoblast differentiation by diverting marrow mesenchymal stem cell (MSC) commitment away from osteoblast and towards adipocyte lineage. Simultaneously, it also promotes recruitment of haematopoietic stem cells (HSCs), driving them towards the osteoclast lineage [18]. In MSCs, the activity of pro-adipocytic PPARγ and pro-osteoblastic RUNX2 is reciprocally regulated at the level of serine phosphorylation. Phosphorylation of S112 in PPARγ and S319 in RUNX2 is mediated by the same mitogen activated protein (MAP) kinases and results in activation of RUNX2 and inhibition of PPARγ, and subsequent differentiation of MSCs towards osteoblasts [19]. Conversely, activation of PPARγ and inhibition of RUNX2 activity requires activation of protein phosphatase 5 (PP5), resulting in dephosphorylation of S112 in PPARγ and S319 in RUNX2, and promoting MSC differentiation toward adipocytes, as opposed to osteoblasts [20]. With respect to bone-resorbing osteoclasts, PPARγ supports their differentiation through direct and indirect mechanisms. In monocytes, PPARγ stimulates osteoclast differentiation through peroxisome proliferator-activated receptor gamma, coactivator 1, beta (PGC-1β)-dependent mechanism and activation of the c-Fos transcription factor, while, in MSCs, PPARγ increases support for osteoclastogenesis by stimulating RANKL production [21, 22]. Using selective modulators and genetic manipulation of PPARγ activity, it has been shown that dephosphorylation of S273, which determines the insulin sensitising activity of this protein, is required for PPARγ’s pro-osteoclastic activity, whereas phosphorylation of S112, which is known to prevent adipose tissue expansion, also correlates with increased bone formation [17]. Thus, these two PPARγ functions are necessary for balanced energy metabolism and insulin sensitivity, while also being inherently tied to processes regulating bone turnover through stimulation of bone formation and bone resorption. On the other hand, insulin resistance and obesity, which at the PPARγ level are associated with the phosphorylation of S273 and dephosphorylation of S112, correlate with decreased bone resorption and bone formation, providing a plausible explanation for attenuated bone turnover in type 2 diabetes.

### The stem cell niche in diabetes

Poor fracture healing and high infection rates constitute a significant clinical issue for individuals with diabetes. The factors discussed above may contribute to poor healing at the cellular level via their effect on the stem cell niche. Delta-like non-canonical Notch ligand 1 (DLK1) represents a common negative regulator of both skeletal stem cell differentiation and glucose metabolism via negative regulation of the osteocalcin–insulin loop [23]. Another strong indication of the link between skeletal stem cell differentiation and glucose metabolism has been provided by a recent study showing that low-grade inflammation in diabetes negatively affects the marrow stem cell niche, resulting in impaired fracture healing [24].

## The skeletal consequences of common diabetic characteristics

### AGEs and bone quality

Type I collagen is a major constituent of bone and provides a structural framework that, upon mineralisation, facilitates the skeleton’s strength. Type I collagen is a fibrillary protein that is organised around a triple-helix motif, causing self-assembly into highly organised fibrils stabilised by enzymatic cross-linking. Besides the natural enzymatic cross-linking, type I collagen may undergo chemical cross-linking, which can occur between ‘free-floating’ sugars in the serum and exposed amino acid residues, leading to post-translational modifications of collagen and, as a result, the production of AGEs. Diabetes predisposes individuals to the accumulation of AGEs in many organs, including bone. With respect to structural properties, the accumulation of AGEs in bone and the formation of intra- and inter-cross-links in collagen fibres decrease bone biomechanical properties by increasing material stiffness. Studies on Zucker diabetic Sprague–Dawley (ZDSD) rats, a rodent model of type 2 diabetes, demonstrated that bone toughness significantly decreased with duration of diabetes despite normal mineralisation [25]. AGEs may also affect bone quality by activating signalling downstream of their receptor (the receptor for advanced glycation end-products [RAGE]). This signalling drives marrow MSC differentiation towards functional osteoblasts, promoting bone formation [26], and positively regulates HSC differentiation into osteoclasts, promoting bone resorption [27]. RAGE may also be produced as a soluble ‘decoy’ receptor that, by binding AGEs, may inhibit AGE–RAGE signalling axes. In type 2 diabetes, it has been shown that low serum levels of soluble RAGE and high serum levels of the AGE pentosidine are indicative of risk for fractures independent of BMD [28].

### Bone vasculature in diabetes

Bone vasculature is critical for bone growth, remodelling and injury healing. It provides a sustained supply of oxygen, nutrients and regulatory factors, and removal of metabolic waste. Up to 10% of cardiac output is distributed to the bone mineral compartment and bone marrow by a complex system of sinusoid and classic capillaries. It is conceivable that the same pathological changes that develop in diabetes in the peripheral vasculature also develop in bone. Thus, diabetic complications, including impairment in endothelium-dependent vasodilation, vascular calcification and defective angiogenesis, may affect the development of osteoblast progenitors from the haematopoietic niche and delivery of osteoblasts (pericytes) and osteoclasts to the bone remodelling unit by capillaries present in Harvesian canals [29]. The reduction in blood flow and impairment in new vessel formation may lead to a decrease in osteoblast formation, decrease in bone remodelling activity and, consequently, a decrease in bone quality and delayed fracture healing.

It has been recently reported that microvascular disease correlates with increased fractures in type 1 diabetes and deficits in the cortical bone in individuals with type 2 diabetes, as compared with individuals without microvascular disease [30, 31]. AGE/RAGE signalling heavily influences vascular calcification, as shown in a number of animal and human studies. Activation of RAGE by AGEs in vascular smooth muscle cells (VSMC) of the peripheral vascular system triggers a signalling cascade involving p38 mitogen activated protein kinase (MAPK), TGF-β and NFκB. Subsequent downregulation of VSMC markers and activation of an osteoblast-like programme, including expression of *Runx2* and osteocalcin and increased enzymatic activity of alkaline phosphatase*,* result in VSMC calcification [32]. Thus, it is possible that vessel calcification in bone may occur by the same mechanism and contribute to decreased blood flow and delivery of progenitors to the bone remodelling unit. Although there is a paucity of animal studies on blood flow and its role in the maintenance of bone homeostasis and impairments in diabetes, it has been demonstrated that the anabolic effect of intermittent parathyroid hormone (PTH) therapy is associated with increased blood flow in bone, suggesting a supportive role of microvasculature during bone formation [33].

### Muscle contribution to skeletal impairment in diabetes

The interaction between bone and skeletal muscle occurs at a biomechanical and physiological level. Muscle action exposes bone to a variety of stimuli, including those generated in exercise. This stimulation can be conveyed in the form of direct force applied to the bone or in the form of released endocrine factors. In effect, both forms of muscle-generated signalling reach cellular components that regulate bone remodelling. Diabetes is often associated with limited regular exercise and a sedentary lifestyle, both of which contribute to metabolic impairment and systemic low-grade inflammation. During exercise, skeletal muscle produces myokines, which are released into the circulation and participate in the regulation of glucose and fatty acid metabolism in an autocrine and endocrine fashion. Among them, IL-6 and irisin have been strongly implicated in coupling energy metabolism with bone metabolism.

The dual effect of the IL-6 cytokine on glucose and bone metabolism has been well documented [34]. Metabolic impairment is accompanied by chronically increased levels of IL-6 in the circulation, which contribute to the development of insulin resistance. In contrast, IL-6 that is released from the muscle in response to exercise has an opposite effect, including reduction in systemic inflammation and increased glucose uptake by the muscle. In bone, IL-6 has a comparable dual effect. Chronically increased levels of IL-6 increase RANKL production and osteoclastic bone resorption. However, mice deficient in IL-6 have low bone mass, reduced osteoblast number and delayed fracture healing [35] suggesting an important role for this cytokine in the maintenance of bone homeostasis. Most recently, it has been demonstrated that IL-6 production in murine exercising muscle is under the control of osteocalcin and that IL-6, through a feed-forward mechanism, increases circulating levels of bioactive osteocalcin [36].

Irisin, a myokine implicated in the ‘beiging’ of fat tissue and in improvements in insulin sensitivity, has been recently shown to have a positive effect on cortical bone [37]. Interestingly, this effect is autonomous in the bone and occurs independent of the effects of irisin on fat tissue metabolism. As demonstrated in vitro, irisin directly signals to MSCs through a specific, not as yet identified receptor and increases the expression of osteoblast gene markers. Together these studies provide a paradigm of the intricate mechanisms for reciprocal regulation of muscle, bone and energy metabolism. Moreover, they provide a plausible scenario for how impairments in energy metabolism may trigger a cascade of signalling events that may affect both bone and muscle.

**Fig. 1:**
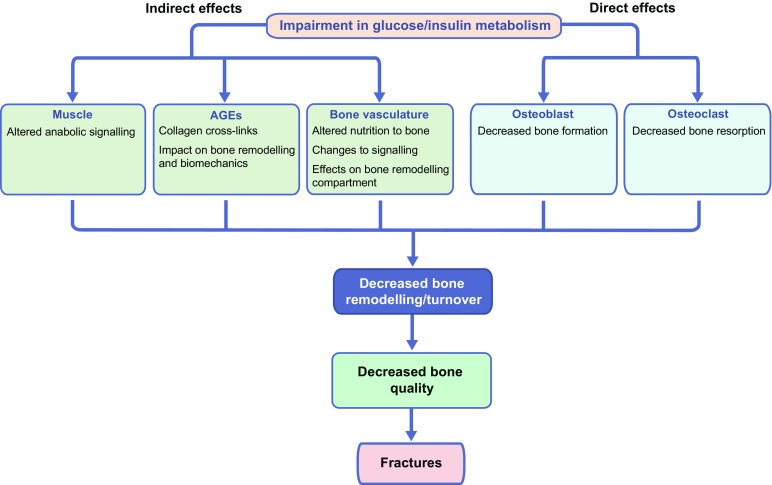
Graphical summation of the effects of diabetes on bone. Impairments in glucose and insulin metabolism have indirect and direct effects on bone quality in individuals with diabetes. Impaired glucose/insulin metabolism may indirectly affect bone by altering skeletal muscle signalling. Furthermore, in metabolic disease, the accumulation of AGEs may trigger pathways that promote collagen cross-linking (altering bone biomechanics) and impact on bone remodelling. Furthermore, the bone vasculature is disturbed with dysregulation in glucose and insulin metabolism, so that the delivery of nutrients and signalling factors (e.g. that regulate vasodilation) to the bone is impaired. The changes in bone vasculature in diabetes results in decreased remodelling activity in the bone. Impairments in glucose and insulin metabolism also directly impact on osteoblast and osteoclast activity, resulting in decreased bone formation and bone resorption. Ultimately, the indirect and direct effects of impaired glucose/insulin metabolism on bone lead to decreased bone remodelling/turnover, decreased bone quality and increased risk of fractures

Fig. 1 summarises the contribution of glucose metabolism, muscle, AGEs and vasculature impairment to decreased bone quality in diabetes.

## Evidence for the effect of glucose-lowering medications for bone health

Glucose-lowering therapies target different aspects of glucose metabolism, including insulin sensitivity (metformin and thiazolidinediones [TZDs]), insulin secretion and bioactivity (sulfonylureas, glucagon-like peptide 1 [GLP-1] analogues, dipeptidyl peptidase 4 [DPP-4] inhibitors and insulin analogues), and modulation of blood glucose levels by either increased excretion (sodium–glucose cotransporter 2 [SGLT2] inhibitors) or delaying its appearance following nutrient digestion (α-glucosidase inhibitors and amylin [amylin is not currently approved for use in the UK]).

### Therapies with either positive or neutral effects on bone

The effects of metformin and sulfonylureas, which account for more than 70% of prescriptions for diabetes [38], are considered as neutral for human bone because there is no correlation between the use of these therapeutic agents and incidence of fractures. However, strong experimental evidence suggests that metformin may be beneficial for bone; in mesenchymal cells, metformin activates a pro-osteoblastic regulatory cascade via RUNX2 and AMP-activated protein kinase (AMPK)/upstream transcription factor 1 (USF1)/small heterodimer partner (SHP) signalling, and inhibits adipogenesis through both AMPK and mechanistic target of rapamycin (mTOR)/p70 S6 kinase (p70^S6K^) signalling [39, 40]. Further, in haematopoietic cells, metformin decreases osteoclast development and prevents macrophage proinflammatory responses to AGEs by decreasing RAGE signalling, potentially decreasing bone marrow support for resorption and proatherosclerotic effects on bone vasculature [39, 41]. To this end, in mice with streptozotocin-induced type 1 diabetes, metformin improved the angiogenic functions of endothelial cells via activation of the AMPK/endothelial nitric oxide synthase (eNOS) pathway [42]. It has also been shown that metformin has a protective effect on bone mass in conditions of oestrogen deficiency [43]. Similarly, some research suggests that glimepiride, a second generation sulfonylurea, may enhance osteoblastic differentiation in high glucose conditions via the phosphoinositide 3-kinase (PI3K)/Akt/eNOS pathway [44] and bone formation in ovariectomised rats [45]. Experimental evidence may differ from clinical observations because the beneficial effects of metformin and sulfonylureas on human bone may be too subtle to be detected in the existing clinical trials, which were not designed to study the effects of these drugs on bone.

Similarly, the skeletal effects of incretin-based therapies, such as GLP-1 analogues, in humans are not clear despite several in vitro and in vivo studies suggesting their beneficial effect. Both osteoblasts and osteoclasts express GLP-1 receptors and treatment of normoglycaemic ovariectomised mice with the receptor agonists, exenatide and liraglutide, increased trabecular, but not cortical bone mass through a combined effect on osteoblast and osteoclast activity [46].

Animal studies on the effect of DPP-4 inhibitors on bone have not shown consistent results.

### Therapies with negative effects on bone

SGLT2 inhibitors form a new class of glucose-lowering medications that have recently been scrutinised for their skeletal effects. There is a paucity of basic research studies on the effect of SGLT2 inhibitors on bone, however one study showed that canagliflozin may exacerbate trabecular bone loss in a streptozotocin-induced murine model of type 1 diabetes [47]. The effects may include increased bone resorption; however the exact mechanism for this outcome has not been demonstrated, raising a question as to whether canagliflozin affects bone directly or systemically [47].

The most notorious drugs that show a negative effect on bone are TZDs, which act as high affinity ligands and activators of PPARγ. It is well documented that clinically approved TZDs, rosiglitazone and pioglitazone, decrease bone mass and increase incidence of fractures, especially in women. As full PPARγ agonists, dephosphorylating both S273 and S112 in the PPARγ protein, TZDs activate pro-adipocytic pathways and suppress pro-osteoblastic programmes in MSCs, while activating pro-osteoclastic programmes in HSCs both directly and via increased RANKL production in mesenchymal cells [22]. In mice, TZDs alter bone remodelling by suppressing bone formation and increasing bone resorption, resulting in decreased trabecular and cortical bone mass. Furthermore, TZD use in mice is associated with massive accumulation of adipocytes in the bone marrow cavity (reviewed in [48]). Recently, it has been shown that TZDs also affect osteocytes, the most abundant cells in bone, which orchestrate the bone remodelling process. In osteocytes, rosiglitazone increases production of pro-osteoblastic Wnt pathway inhibitors (sclerostin and dickkopf-related protein 1 [DKK1]) and of the pro-osteoclastic cytokine RANKL [17, 49].

In conclusion, the most common therapies, metformin and sulfonylurea, are safe for use in relation to their effect on bone, whereas less frequent therapies, such as TZDs and possibly SGLT2, may increase risk of fractures. Yet, with respect to bone disease in diabetes, the ideal glucose-lowering therapy would prevent fractures by supporting bone remodelling and, hence, increasing bone quality. There is no evidence that any existing glucose-lowering therapies provide such benefits to bone. However, new research offers a paradigm of glucose-lowering drugs that may increase bone quality in individuals with diabetes by increasing bone turnover. In mice, a novel insulin sensitising agent, SR10171, which is an optimised derivative of 2-phenoxypropanoic acid and acts as an inverse agonist for PPARγ and a weak agonist for PPARα, increases bone mass and bone turnover by modulating the activity of osteoblasts, osteoclasts and osteocytes [17]. Thus, SR10171 may be considered a prototype drug that may target both insulin resistance and bone disease in diabetes.

## Perspectives and future directions

In diabetes, bone disease has a complex pathology, hallmarked by increased fractures independent of BMD. This complexity results from the contribution of multiple physiological processes that are impaired in diabetes and contribute simultaneously to the negative effect of diabetes on bone and energy metabolism. There is an array of aspects of bone disease in diabetes that future studies must address, including the presence of biomarkers for the prediction of fracture risk and improvements in fracture healing, and multi-level pharmacological effects of glucose-lowering therapies on bone. There is also a need to develop a comprehensive animal model that would accurately reflect human bone disease in diabetes, including the intricacies of inter-organ communication.

## Electronic supplementary material

ESM 1 (PPTX 144 kb)

## Abbreviations

AGEs: Advanced glycation end-products
AMPK: AMP-activated protein kinase
BMD: Bone mineral density
DIO: Diet-induced obesity
DPP-4: Dipeptidyl peptidase 4
eNOS: Endothelial nitric oxide synthase
GLP-1: Glucagon-like peptide 1
HSCs: Haematopoietic stem cells
MSC: Mesenchymal stem cell
PPAR: Peroxisome proliferator activated receptor
RAGE: Receptor for advanced glycation end-products
RANKL: Receptor activator of nuclear factor κB ligand
RUNX2: Runt-related transcription factor 2
SGLT2: Sodium–glucose cotransporter 2
TZDs: Thiazolidinediones
VSMC: Vascular smooth muscle cells
