# Supplementary material for: Diabetes, bone and glucose-lowering agents: basic biology
Source: Diabetologia. 2017 Apr 22;60(7):1163–9. doi: 10.1007/s00125-017-4269-4 (PMC5487688; doi:10.1007/s00125-017-4269-4)
Supplement: Supplementary file 1 — (PPTX 144 kb) [file 125_2017_4269_MOESM1_ESM.pptx]

## Slide 1
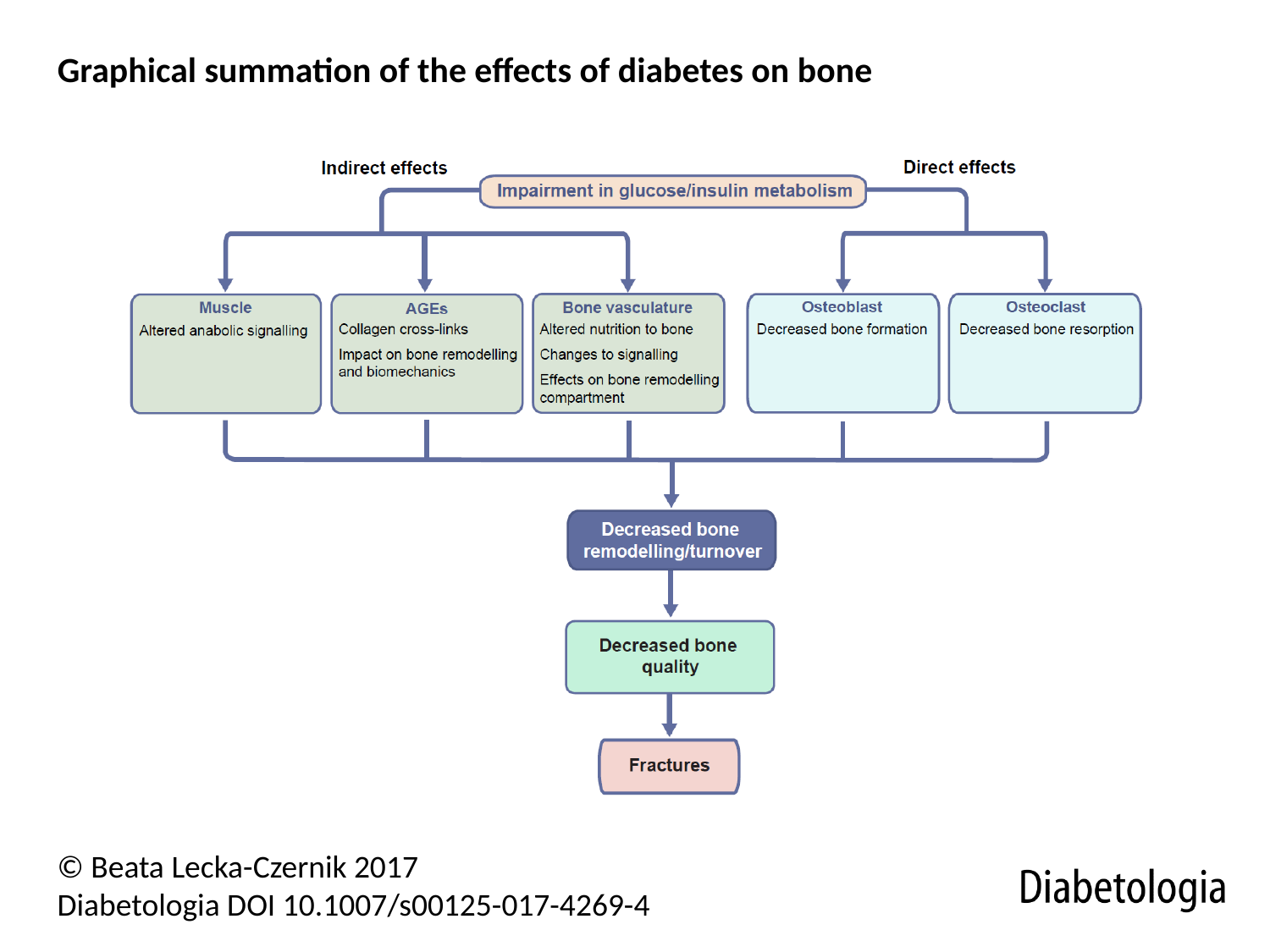

Graphical summation of the effects of diabetes on bone
© Beata Lecka-Czernik 2017
Diabetologia DOI 10.1007/s00125-017-4269-4
